# Global Research Trends on Infertility and Psychology From the Past Two Decades: A Bibliometric and Visualized Study

**DOI:** 10.3389/fendo.2022.889845

**Published:** 2022-07-12

**Authors:** Hongkun Zhu, Lingli Shi, Rong Wang, Lijuan Cui, Jiahui Wang, Mengyu Tang, Haiqing Qian, Minggang Wei, Lihong Wang, Huifang Zhou, Wenting Xu

**Affiliations:** ^1^ Nanjing University of Chinese Medicine, Nanjing, China; ^2^ Department of Reproduction, Zhangjiagang TCM Hospital Affiliated to Nanjing University of Chinese Medicine, Suzhou, China; ^3^ Department of Laboratory Medicine, Zhangjiagang TCM Hospital Affiliated to Nanjing University of Chinese Medicine, Suzhou, China; ^4^ Department of Pathology, Zhangjiagang TCM Hospital Affiliated to Nanjing University of Chinese Medicine, Suzhou, China; ^5^ Department of Traditional Chinese Medicine, The First Affiliated Hospital of Soochow University, Suzhou, Jiangsu, China; ^6^ Department of Gynaecology, Affiliated Hospital of Nanjing University of Chinese Medicine, Nanjing, China

**Keywords:** infertility and psychology, bibliometric analysis, VOSviewer, web of science, research trends, hot spots

## Abstract

**Objectives:**

The aim of this study was to evaluate the global scientific output of research on infertility and psychology; explore the current status and trends in this field through the cooperation of authors, countries, and institutions; shed light on the direction of clinical infertility research in the future, and provide inspiration for targeted diagnosis and treatment of infertility.

**Methods:**

Research publications on infertility and psychology from the past two decades were retrieved from the Web of Science Core Collection (WoSCC). Bibliometric analyses were performed using VOSviewer software and the bibliometrix R package. Network maps were generated to evaluate the collaborations between different authors, countries, institutions, and keywords.

**Results:**

A total of 151 articles related to the study of infertility and psychology were identified. We observed a gradual increase in the number of publications from 2001 to 2021, and the trend has been relatively stable in the past eight years. Human Reproduction (England), as the leading journal publishing the most papers (29 articles), was cited in the most journals (1208 times). Boivin J was the most prolific author (16 articles), with the largest number of citations (890 times) and the highest h-index (14) during the past decades. Boivin J was also the leader with the highest publication frequency and more active cooperation with other top authors. The United Kingdom (34 papers) and Cardiff University (25 articles) contributed the most publications and were the leading contributors in this field. Active cooperation between countries and between institutions was observed, and analyses of articles and references were also shown. The main hot topics included matters related to women (39 times), *in-vitro* salt (31 times), infertility (30 times), couples (25 times), and impact (24 times).

**Conclusion:**

Our study results provide a comprehensive overview of the development of scientific literature, allowing relevant authors and research teams to recognize the current research status in this field. At the same time, infertility and psychology may soon become hotspots and should be closely monitored.

## 1 Introduction

Infertility is the inability to conceive after at least one year of unprotected intercourse. Women over 35 years of age can shorten this period to 6 months owing to decreased fertility (1). Many factors can give rise to infertility, mainly the following three: male factors, female factors, or both. Male and female factors were present in 35% of couples in a 2019 study (2). Male infertility can be attributed to testicular or ejaculatory dysfunction, hormonal disturbances, or genetic disorders, while female infertility can be attributed to ovarian dysfunction, tubal obstruction, or an abnormal uterine structure (2). With the improvement in financial ability and the availability of multiple birth plans, more people have made pregnancy plans, so infertility diagnosis has also increased relatively. According to a 2019 CDC survey, 12.7% of women between the ages of 15 and 49 received infertility services, most women sought medical help to become pregnant (9.5%), 6.7% sought basic medical advice, 5.8% of the population was only tested for infertility, and assisted reproductive technology (ART) was the least sought service (0.6%) (2).

The causes of infertility are not only medical but also psychosocial. Although male and/or female factors are associated with infertility, the specific causes of infertility in a specific couple cannot be identified. Infertility can be associated with different clinical symptoms for females, such as menstrual disorder, obesity, hypertrichosis, and seborrheic alopecia, which can seriously affect the patient’s quality of life and alter their appearance. This situation can make patients feel anxious or depressed, have low self-esteem, go into self-isolation, or experience other negative emotions, which further affect the quality of life and break the neuroendocrine balance. Studies have shown that infertility causes as much psychological stress as cancer or heart disease (3). The current treatment methods for infertility mainly include drug-induced ovulation therapy, hysteroscopic surgery, intrauterine insemination (IUI), *in vitro* fertilization and embryo transfer (IVF-ET), and third-party-assisted ART (with gamete donors) (4). Whether a drug treatment or surgical treatment, there is a certain treatment cycle, especially in IVF-ET, which is the core technology of ART. IVF-ET is an expensive and complicated process. The lack of correct understanding of related assisted reproductive technology can bring a certain level of public opinion pressure, have different degrees of psychological impact on patients, and produce negative emotions. This kind of psychological stress and infertility can cause and affect each other and eventually form a vicious circle (5). Moreover, new technologies tend to increase the treatment confidence of infertile patients and give them a stronger willingness to give birth. The willingness has also aggravated the psychological pressure on patients to conceive to a certain extent (6).

Previous studies on infertility have focused on physiology, pathology, and treatment. The research on the impact of psychological factors and treatment methods has been insufficient. This study aimed to conduct a statistical analysis of relevant literature through the global scientific output and the cooperation of authors, countries, and institutions. We found that psychology is strongly associated with infertility. This study shed light on the direction of clinical infertility research in the future and provides inspiration for the targeted diagnosis and treatment of infertility.

## 2 Research Methodology

### 2.1 Assembling

This study collects bibliometric data on infertility and psychology research for its review. In order to explore the influence of psychology on infertility, we selected “infertility” and “psychology” as the search terms based on previous literature retrieval methods. This study conducted a search for articles using the aforementioned search string in the”article title, abstract, and keywords” on the Web of science core collection (WoSCC). The WoSCC covers a considerable amount of high-quality scientific literature in the biomedical, natural, and social sciences, and it was used as the main data source. It is regarded as one of the most widely accepted and suitable databases for the bibliometric analysis of scientific publications (7). At the same time, it also contains the cited references in each publication, prepares the indexes according to the cited author, source and year of publication, and establishes the world’s most influential and authoritative citation index database. Through a unique citation index, the evolution of research content and research direction can be understood without being limited by keyword changes.

### 2.2 Arranging

**Figure 1:**
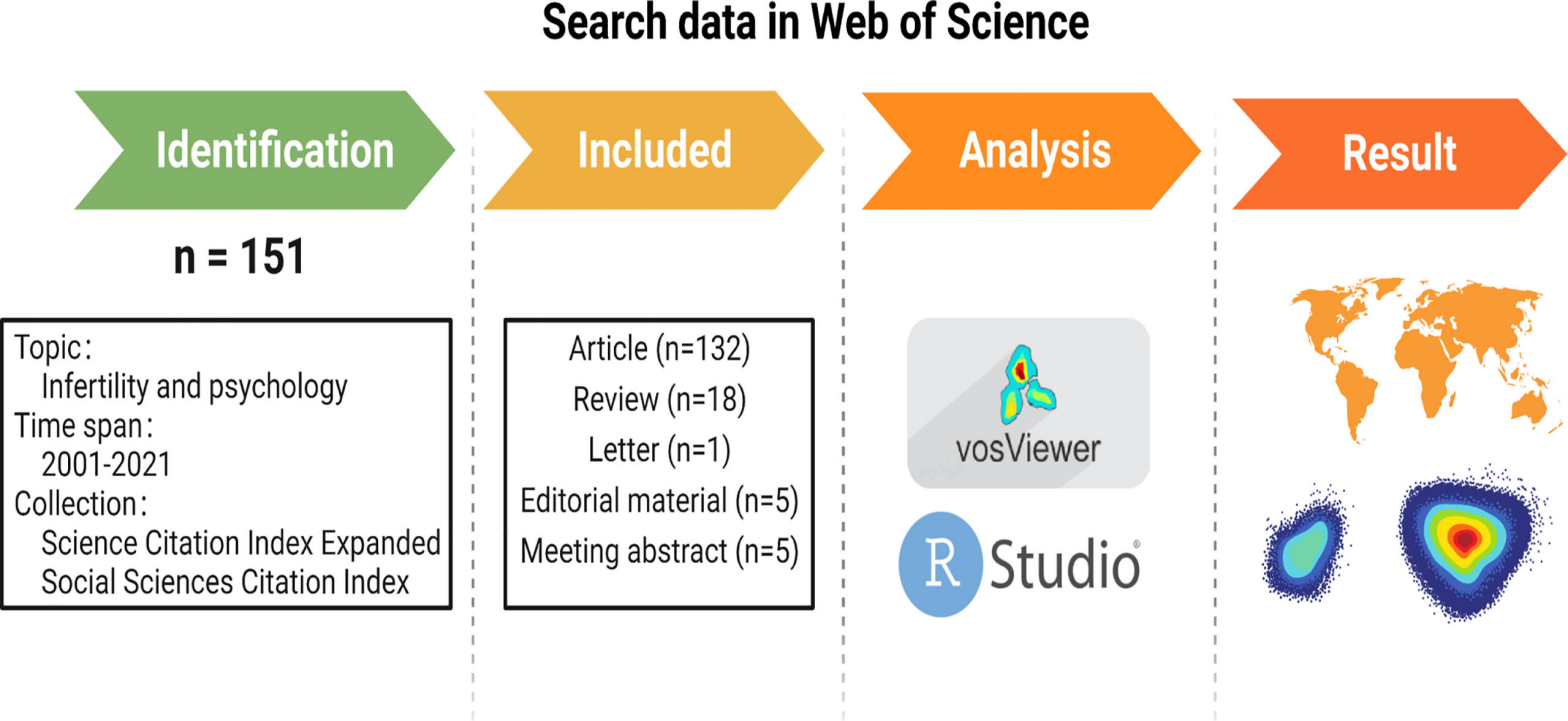
Flow diagram of the included papers created with BioRender.com.

We expanded research literature retrieval, analysis, retrieval rules, and measurement methods through the WoSCC (**Figure 1**). Then, 151 kinds of literature with the subject words “infertility” and “psychology” in the English language were selected from the Science Citation Index (SCI-Expanded) and the Social Sciences Citation Index from January 2001 to November 2021. All the documents published in the journal until 16 November 2021 were adopted. There were 151 documents in total, including articles, editorial materials, letters, meeting abstracts, and reviews. All the retrieved papers from the WoSCC, including the titles, keywords, author information, abstracts, and references, were downloaded and saved in the Bibtex file format.

### 2.3 Assessing

To assess the final corpus of 151 articles on infertility and psychology, this study adopts a bibliometric analysis approach for its review. We imported all the Bibtex files obtained above into the Biblioshiny application by using R software (version 4.0.2), Rstudio software (version 1.3.959), and the bibliometrix R package (https://www.bibliometrix.org). We completed the conversion of the original data to the data frameset. These files were imported into Microsoft Excel 2019 for further data processing. Two researchers independently performed the literature selection, data extraction, and analysis to ensure the reliability of the results. Data extracted from the selected articles include the general information about the annual number of publications, citation frequency, original countries, authors, journals, and institutions. The author’s publication-quality was assessed based upon metrics that included the number of publications, citations in the research area, and publication h-index value. The above kinds of literature underwent data analysis and visualization using VOSviewer (Version 1.6.16, Leiden University, the Netherlands).VOSviewer was used to create network visualization maps to analyze the collaborative relationships between countries/regions, institutions, and authors of highly cited references. In addition, VOSviewer can classify keywords with high co-occurrence frequencies into several clusters and simultaneously color them by time course. Co-occurrence analysis identifies research hotspots and trends.

## 3 Results

### 3.1 Publication Output and Temporal Trend

**Figure 2:**
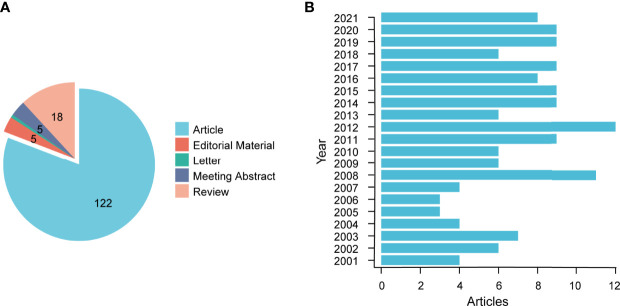
**(A)** Statistical chart of literature types **(B)** Global trend of annual publications related to infertility and psychology research from 2001 to 2021.

According to the above retrieval methods and data processing, a total of 151 publications were obtained from the WoSCC, which were published from 2001 to 2021. Broadly speaking, the number of publications was not large, which indicates that there is still a lack of research attention to infertility and psychology. Among them, there were 122 papers (80.79%), 18 reviews (11.92%), five editorial materials (3.31%), five meeting abstracts (3.31%), and one letter (0.66%). The literature category distribution can be found in **Figure 2A**. Since 2001, the publications of related literature on “infertility” and “psychology” showed a fluctuating upward trend, reaching two peaks in 2008 and 2012, and 2012 (12 publications, 7.95%) was the most prolific year for publications. The annual publication output was stable, at 8 in the next eight years (**Figure 2B**).

### 3.2 Analysis of Leading Journals and Cited Journals

**Figure 3:**
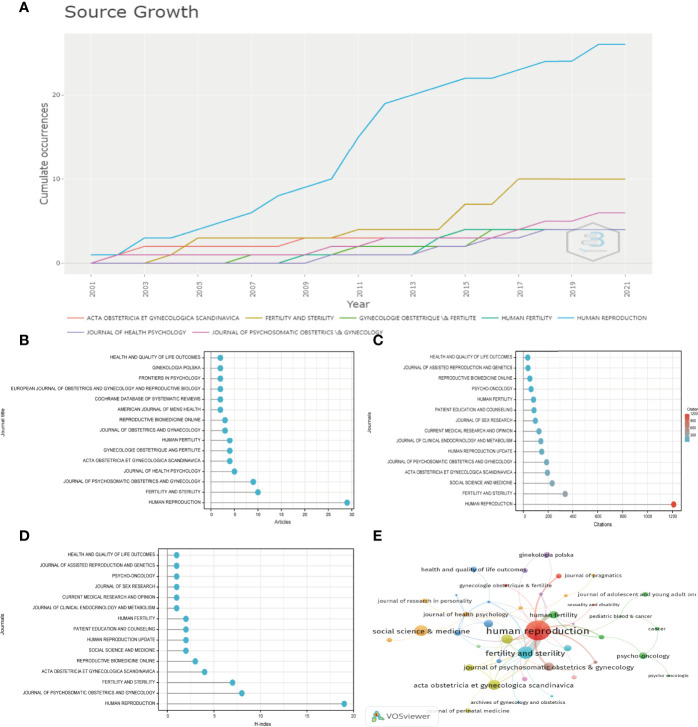
**(A)** Growth trends in the publication quantity of the top 7 journals in infertility and psychology research from 2001 to 2021 **(B–D)** Total number of publications, sum of total citations, and H-index of top 15 journals in this field. **(E)** Citation map of journals on infertility and psychology research generated by the VOS viewer. Each node represents a journal, and node size indicates the number of publications. The connection between the nodes represents a citation relationship, and the thickness of the lines indicates citation strength.

In total, 75 academic journals published papers about infertility and psychology. **Table 1** and **Figure 3B** present the top 15 most popular journals contributing to articles on infertility and psychological topics, and it also shows the countries of origin and impact factor of the top 15 journals. Human Reproduction (England) was the leading journal, publishing the most papers (29 articles), followed by Fertility and Sterility (10 articles, United States), Journal of Psychosomatic Obstetrics and Gynecology (nine articles, Netherlands), Journal of Health Psychology (five articles, England), Acta obstetricia et Gynecologica Scandinavica (four articles, Denmark), Gynecologie Obstetrique and Fertilite (four articles, France), Human Fertility (four articles, England), Journal of Obstetrics and Gynaecology (three articles, England), Reproductive Biomedicine Online (three articles, England), American Journal of Men’s Health (two articles, United States), Cochrane database of systematic reviews (two articles, England), European Journal of Obstetrics and Gynecology and Reproductive Biology (two articles, Netherlands), Frontiers in Psychology (two articles, Switzerland), Ginekologia Polska (two articles, Poland), and Health and Quality of Life Outcomes (two articles, England). The impact factor (IF) of Human Reproduction (the most published journal) was 6.918 (2020), and the H-index was 19, putting it in the first place (**Figure 3D**). Among the top 15 journals, the highest IF was 9.289 (2020), the Cochrane database of systematic reviews from England. This journal had a small number of articles, with only two articles. The volume of literature in these journals has been increasing year by year. Human Reproduction had the largest increase among these journals (**Figure 3A**). As a result, the psychological aspect of infertility is attracting increasing attention, which can provide suggestions for future research. The presented data are consistent with the current model of bio-psycho-social medicine.

**Table 1:** Top 15 journals in the field of infertility and psychology research ranked by publication number.

Rank	Journal title	Articles	Impact factor(2020)	Region
1	Human reproduction	29	6.918	England
2	Fertility and Sterility	10	7.329	USA
3	Journal of Psychosomatic Obstetrics and Gynecology	9	2.949	Netherlands
4	Journal of Health Psychology	5	3.231	England
5	Acta Obstetricia Et Gynecologica Scandinavica	4	3.636	Denmark
6	Gynecologie Obstetrique Ang Fertilite	4	0.429(2016)	France
7	Human Fertility	4	2.767	England
8	Journal of Obstetrics and Gynaecology	3	1.246	England
9	Reproductive Biomedicine Online	3	3.828	England
10	American Journal of Mens Health	2	2.804	USA
11	Cochrane Database of Systematic Reviews	2	9.289	England
12	European Journal of Obstetrics and Gynecology and Reproductive Biology	2	2.435	Netherlands
13	Frontiers in Psychology	2	2.988	Switzerland
14	Ginekologia Polska	2	1.232	Poland
15	Health and Quality of Life Outcomes	2	3.186	England

**Figures 3C, E** present the top 15 cited journals on infertility and psychology research. Human Reproduction was cited in the most journals (1208 times), followed by Fertility and Sterility (341 times). The highest citation frequency of this journal may be due to it being the journal with the highest H-index and the highest number of articles. This also provides a direction for us to find relevant articles in the future. Social Science and Medicine ranked third, with 232 citations. The fourth and fifth, with more than 150 citations, were Acta Obstetricia et Gynecologica Scandinavica (194 times) and Journal of Psychosomatic Obstetrics And Gynecology (187 times), respectively.

### 3.3 Analysis of Authors

**Figure 4:**
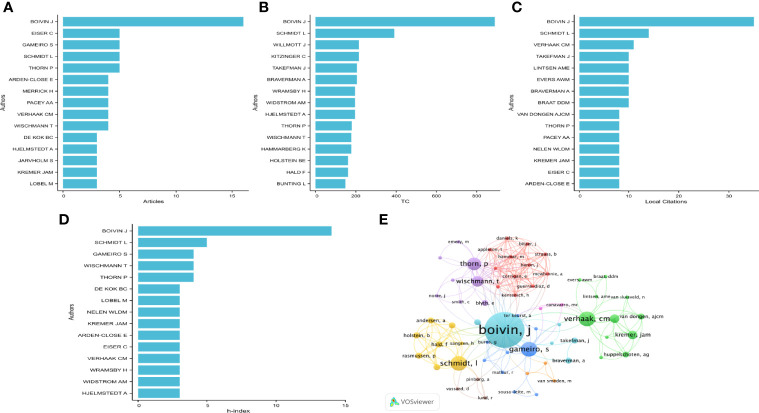
Analysis of authors. **(A)** Number of publications from different authors(Top15). **(B, C)** Total citations and local citations in the research filed from different authors(Top15). **(D)** H-index of publications from different authors(Top15). **(E)** Network map of co-authorship between authors with more than one publications.

In terms of the number of published papers, Boivin J was the most prolific author, with 16 articles (10.60%), followed by Eiser C, Gameiro S, Schmidt L, and Thorn P, each with five articles (3.31%). There were five authors who published four articles, including Arden-close E, Merrick H, Pacey AA, Verhaak CM, and Wischmann T (**Figure 4A**). In terms of citation frequency, Boivin J published the most articles and was cited the most, up to 890 times. His articles were also cited mostly in local citations (cited 35 times, **Figure 4C**). Schmidt L was the second most frequently cited author after Boivin J, with 390 citations. Schmidt l was followed by Kitzinger C (cited 215 times), Willmott J (cited 215 times), Braverman A (cited 205 times), Takefman J (cited 205 times), Hjelmstedt A (cited 196 times), Widstrom AM (cited 196 times), Wramsby H (cited 196 times), and Thorn P (cited 180 times) (**Figure 4B**).

H-index was used to evaluate the influence of authors. The most influential author, Boivin J, had an h-index of 14, followed by Schmidt L, with an h-index of 5. Moreover, authors with an h-index of 4 included Thorn P, Wischmann T, and Gameiro S (**Figure 4D**). We analyzed a total of 487 authors who had co-authored in more than one publication. It can be seen from **Figure 4E** that there were mainly three research teams in author cooperation, of which author Boivin J was the leader, with the highest frequency and the closest cooperation with other authors among the research teams (**Figure 4E**).

### 3.4 Distribution of Institutions

**Figure 5:**
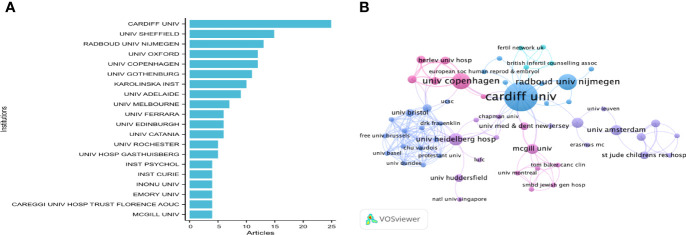
**(A)** The top 20 productive institution on the research of infertility and psychology. **(B)** The institutions collaboration network of research on infertility and psychology; the font size of each institution’s name represents the number of articles in the institutions. The thickness of the curved connecting line represents the collaborative intensity between institutions.

According to the statistics, a total of 251 institutions were involved in this field. The 20 institutions with the highest number of articles were obtained (**Figure 5A**). Cardiff University (25 records, 4.67% of all articles) contributed the most publications, followed by the University of Sheffield (15,2.80%), Radboud University Nijmegen (13, 2.43%), the University of Copenhagen (12, 2.24%), Oxford University (12, 2.24%) Gothenburg (11, 2.06%), and Karolinska Institute (10, 1.87%). Institutions that published more than four articles and fewer than ten articles were the University of Adelaide (9, 1.68%) and the University of Melbourne (7, 1.31%). University Catania, University of Edinburgh, and University Ferrara published six articles (1.12%). University Hospital Gasthuisberg and University of Rochester published five articles (0.93%). McGill University, Careggi University Hospital Trust Florence AOUC, Emory University, Inonu University, Institute Curie, and Institute Psychology published four articles (0.75%).

To reveal potential collaborations among institutions, we used the VOS viewer to conduct a co-authorship analysis in terms of institutions. Among 251 co-authored institutions publishing more than one article (**Figure 5B**), the five institutions with the highest total link strength were Cardiff University (total link strength 26), Heidelberg University Hospital (20), University Wales College Cardiff (19), University of Bristol (15), and Centre Hospitalier Universitaire Vaudois (14). Among these institutions, universities were the main contributors, followed by hospitals. Thus, the focus of infertility and psychology research has gradually transferred from the clinical area to the teaching area and become gradually known to the public.

### 3.5 Analysis of Leading Countries

**Figure 6:**
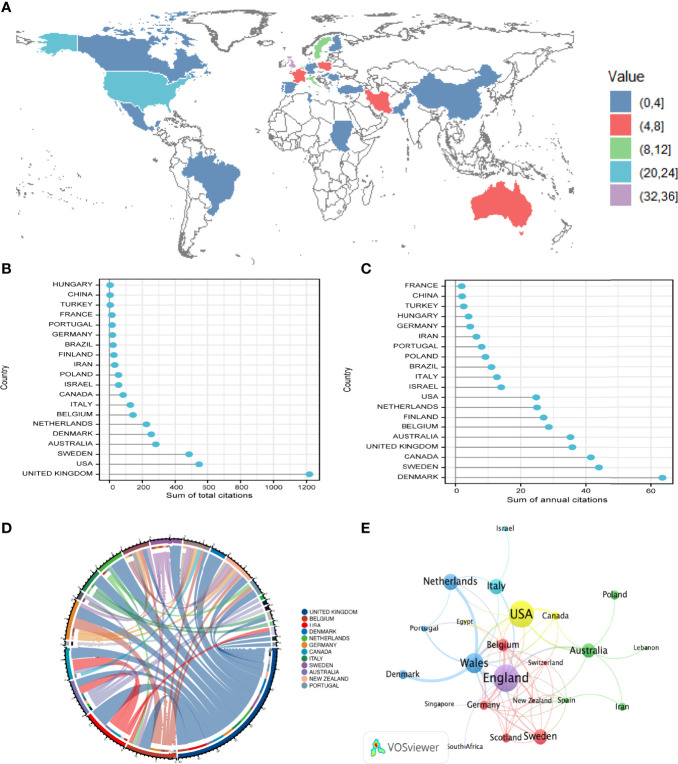
**(A)** World map displaying the global distribution of infertility and psychology research. Different corresponding authors’countries were denoted with different colors based on the number of articles published. **(B, C)** Total citations and annual citations of related articles from top 20 countries. **(D)** Distribution and international cooperation of countries that are involved in infertility and psychology research. The thickness of the line reflects the frequency of the cooperation. The thicker the line, the stronger the cooperation. **(E)** Network map of co-authorship between countries with more than one publications generated by the VOS viewer.Each node represents a country, and node size indicates the number of publications. The connection between the nodes represents a citation relationship, and the thickness of the lines indicates citation strength (weights on the TLS).

The distribution of published articles is shown on a world map by R (**Table 2**; **Figure 6A**). Colors on the map represent different density values. A total of 20 countries contributed to publications in this field. The countries with the most publications were mainly European and the United States. The United Kingdom contributed the highest number of articles (34, 23.94% of all articles), followed by the United States (22, 15.49%), Sweden (11, 7.75%), and Italy (10, 7.04%). Countries with fewer than ten articles were the Netherlands (9, 6.34%); Australia and France (8, 5.63%); Poland (6, 4.23%); Belgium and Iran (5, 3.52%); Denmark, Germany, and Israel (4, 2.82%); and Brazil, Canada, China, Portugal, Turkey, Finland, and Greece publishing the fewest, with only one or two articles (**Table 2**). The United Kingdom had the largest number of articles published by a single country (23 articles), followed by the United States (19 articles), and all other countries had fewer than ten articles.

**Table 2:** Total number of publications, single country publications, and multiple country publications of top 20 Countries by corresponding authors in this field.

Rank	Country	Articles	Freq	SCP	MCP	MCP_Ratio
1	United Kingdom	34	0.22819	23	11	0.324
2	USA	22	0.14765	19	3	0.136
3	Sweden	11	0.07383	9	2	0.182
4	ITALY	10	0.06711	8	2	0.2
5	Netherlands	9	0.0604	7	2	0.222
6	Australia	8	0.05369	5	3	0.375
7	France	8	0.05369	8	0	0
8	Poland	6	0.04027	5	1	0.167
9	Belgium	5	0.03356	4	1	0.2
10	Irann	5	0.03356	3	2	0.4
11	Denmark	4	0.02685	2	2	0.5
12	Germany	4	0.02685	3	1	0.25
13	Israel	4	0.02685	3	1	0.25
14	Brazil	2	0.01342	2	0	0
15	Canada	2	0.01342	1	1	0.5
16	China	2	0.01342	2	0	0
17	Portugal	2	0.01342	2	0	0
18	Turkey	2	0.01342	2	0	0
19	Finland	1	0.00671	1	0	0
20	Greece	1	0.00671	1	0	0

SCP, single country publications; MCP, multiple country publications

Studies from the United Kingdom had the highest number of citations (1,218 citations), followed by those from the United States (545 citations), Sweden (484 citations), Australia (282 citations), Denmark (254 citations), Netherlands (225 citations), Belgium (143 citations), Italy (127 citations), Canada (83 citations), Israel (56 citations), Poland (55 citations), Iran (32 citations), Finland (27 citations), Brazil (22 citations), Germany (18 citations), Portugal (16 citations), France (15 citations), and Turkey (five citations) (**Figure 6B**). China and Hungary had the lowest number of citations, with four citations. According to the analysis of the countries with the highest annual citation frequency (**Figure 6C**), although the articles of British scholars were the most frequently cited in total, they ranked fourth in the annual citation frequency (35.82), and Denmark ranked first in the annual citation frequency (63.50), followed by Sweden (44) and Canada (41.50).

In terms of international cooperation, through the analysis of the countries of origin of the corresponding authors of the articles, the United Kingdom had the most precise cooperation with various countries (**Figure 6D**), with a total of 40 cooperation articles, including five cooperation articles with the United States; four cooperation articles with Denmark and Netherlands; three cooperation articles with Belgium, Canada, Germany, Italy, and Sweden; and two cooperation articles with Australia, New Zealand, and Portugal. The United Kingdom cooperated with China, Egypt, Singapore, South Africa, Spain, and Switzerland, the number of articles was one. A total of 36 countries with more than one publication in the field were analyzed in the co-authorship analysis (**Figure 6E**). The five countries with the highest total link strength were Wales (total link strength 29 times), England (23), Belgium (15), the United States (14), and Germany (13). Furthermore, Wales cooperated most closely with Denmark and Netherlands. These countries published more articles.

### 3.6 Citation and Reference Analyses

**Figure 7:**
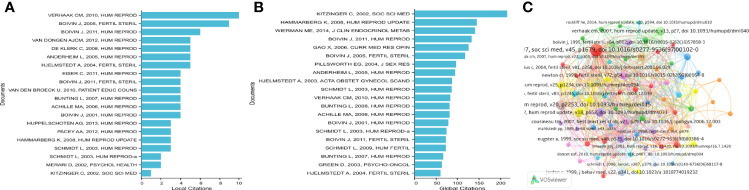
**(A, B)** Top 20 local and global citation analysis of documents on infertility and psychology research. **(C)** Networkmap of co-citation analysis of references with more than five citations.

We conducted a statistical analysis of 151 articles and found that the citation analysis showed that seven documents had more than five citations (**Table 3**), “Who Is at Risk of Emotional Problems and How Do You Know? Screening of Women Going for IVF Treatment” was cited ten times in local citations (**Figure 7A**). In addition, that article was not the most frequently cited article in global citations (**Figure 7B**). Kitzinger C’s article, “The Thief of Womanhood: Women’s Experience of Polycystic Ovarian Syndrome,” published in SOC SCI MED in 2002, was cited up to 215 times, and the current number of local citations was 1, ranking 20th.

**Table 3:** Top 20 citation analysis of documents on infertility and psychology research.

Document	Year	Title	Local Citations	Global Citations	LC/GC Ratio (%)	Normalized Local Citations	Normalized Global Citations
VERHAAK CM, (8), HUM REPROD	2010	WHO IS AT RISK OF EMOTIONAL PROBLEMS AND HOW DO YOU KNOW? SCREENING OF WOMEN GOING FOR IVF TREATMENT	10	84	11.90	4.29	3.13
BOIVIN J, (9), FERTIL STERIL	2005	INFERTILITY-RELATED STRESS IN MEN AND WOMEN PREDICTS TREATMENT OUTCOME 1 YEAR LATER	9	118	7.63	1.93	1.66
BOIVIN J, (10), HUM REPROD	2011	THE FERTILITY QUALITY OF LIFE (FERTIQOL) TOOL: DEVELOPMENT AND GENERAL PSYCHOMETRIC PROPERTIES	6	133	4.51	3.38	3.15
HJELMSTEDT A (11), FERTIL STERIL	2004	EMOTIONAL ADAPTATION FOLLOWING SUCCESSFUL IN VITRO FERTILIZATION	5	60	8.33	4.00	1.35
ANDERHEIM L, (12), HUM REPROD	2005	DOES PSYCHOLOGICAL STRESS AFFECT THE OUTCOME OF IN VITRO FERTILIZATION?	5	95	5.26	1.07	1.34
DE KLERK C, (13), HUM REPROD	2008	LOW NEGATIVE AFFECT PRIOR TO TREATMENT IS ASSOCIATED WITH A DECREASED CHANCE OF LIVE BIRTH FROM A FIRST IVF CYCLE	5	46	10.87	5.00	1.29
VAN DONGEN AJCM, (14), HUM REPROD	2012	FEASIBILITY OF SCREENING PATIENTS FOR EMOTIONAL RISK FACTORS BEFORE IN VITRO FERTILIZATION IN DAILY CLINICAL PRACTICE: A PROCESS EVALUATION	5	15	33.33	6.00	0.77
BOIVIN J, (15), HUM REPROD	2001	GUIDELINES FOR COUNSELLING IN INFERTILITY: OUTLINE VERSION	4	78	5.13	4.00	2.64
ACHILLE MA, (16), HUM REPROD	2006	FACILITATORS AND OBSTACLES TO SPERM BANKING IN YOUNG MEN RECEIVING GONADOTOXIC CHEMOTHERAPY FOR CANCER: THE PERSPECTIVE OF SURVIVORS AND HEALTH CARE PROFESSIONALS	4	82	4.88	3.00	1.16
BUNTING L (17), HUM REPROD	2007	DECISION-MAKING ABOUT SEEKING MEDICAL ADVICE IN AN INTERNET SAMPLE OF WOMEN TRYING TO GET PREGNANT	4	66	6.06	4.00	2.51
VAN DEN BROECK U, (18), PATIENT EDUC COUNS	2010	COUNSELLING IN INFERTILITY: INDIVIDUAL, COUPLE AND GROUP INTERVENTIONS	4	48	8.33	1.71	1.79
BOIVIN J, (10), FERTIL STERIL	2011	THE FERTILITY QUALITY OF LIFE (FERTIQOL) TOOL: DEVELOPMENT AND GENERAL PSYCHOMETRIC PROPERTIES	4	72	5.56	2.25	1.71
EISER C, (19), HUM REPROD	2011	THE LEGACY OF SPERM BANKING: HOW FERTILITY MONITORING AND DISPOSAL OF SPERM ARE LINKED WITH VIEWS OF CANCER TREATMENT	4	34	11.76	2.25	0.81
SCHMIDT L (20), HUM REPROD	2003	PATIENTS’ ATTITUDES TO MEDICAL AND PSYCHOSOCIAL ASPECTS OF CARE IN FERTILITY CLINICS: FINDINGS FROM THE COPENHAGEN MULTI-CENTRE PSYCHOSOCIAL INFERTILITY (COMPI) RESEARCH PROGRAMME	3	87	3.45	4.20	1.67
HAMMARBERG K, (21), HUM REPROD UPDATE	2008	PSYCHOLOGICAL AND SOCIAL ASPECTS OF PREGNANCY, CHILDBIRTH AND EARLY PARENTING AFTER ASSISTED CONCEPTION: A SYSTEMATIC REVIEW	3	145	2.07	3.00	4.06
PACEY AA, (22), HUM REPROD	2012	MONITORING FERTILITY (SEMEN ANALYSIS) BY CANCER SURVIVORS WHO BANKED SPERM PRIOR TO CANCER TREATMENT	3	23	13.04	3.60	1.18
HUPPELSCHOTEN AG, 23, HUM REPROD	2013	DIFFERENCES IN QUALITY OF LIFE AND EMOTIONAL STATUS BETWEEN INFERTILE WOMEN AND THEIR PARTNERS	3	56	5.36	6.00	3.43
MERARI D, (24), PSYCHOL HEALTH	2002	EMOTIONAL REACTIONS AND ATTITUDES PRIOR TO IN VITRO FERTILIZATION: AN INTER-SPOUSE STUDY	2	25	8.00	4.00	0.43
SCHMIDT L (25), HUM REPROD-a	2003	HIGH RATINGS OF SATISFACTION WITH FERTILITY TREATMENT ARE COMMON: FINDINGS FROM THE COPENHAGEN MULTI-CENTRE PSYCHOSOCIAL INFERTILITY (COMPI) RESEARCH PROGRAMME	2	74	2.70	2.80	1.42
KITZINGER C (26), SOC SCI MED	2002	`THE THIEF OF WOMANHOOD’: WOMEN’S EXPERIENCE OF POLYCYSTIC OVARIAN SYNDROME	1	215	0.47	2.00	3.71

**Table 4** enumerates the basic information for the papers among the top 20 most cited. These highly cited studies were published between 1991 and 2012, and 11 studies were published before 2000. The most cited reference was GREIL AL’s book, Infertility and Psychological Distress: A Critical Review of the Literature, which was a literature review of the psychosocial effects of infertility, published in SOC SCI MED in 1997. It was cited 24 times. Verhaak CM (2005) wrote the second most highly cited paper, with 17 citations. The two papers written by Gameiro S (2012) and Verhaak CM (2007) ranked third, with 14 citations. The fourth position was occupied by Boivin J (2007), with 13 citations, followed by Slade P (1997) (13 citations), Eugster A (1999) (11 citations), and Newton CR (1999) (11 citations). The references written by Boivin J (2011), Cousineau TM (2007), Greil AL (2010), Jordan C (1999), and Verhaak CM (2010) were cited ten times. As shown in **Figure 7C**, the co-citation network consisted of 5000 cited references and could be shown as 78 cited references, which had more than five citations of a cited reference.

**Table 4:** The top 20 references with the strongest citation bursts in the co-citation network on infertility and psychology research.

Cited References	Title	DOI	Citations
GREIL AL, (27), SOC SCI MED, V45, P1679	Infertility and psychological distress: A critical review of the literature	10.1016/S0277-9536(97)00102-0	24
VERHAAK CM, (28), HUM REPROD, V20, P2253	A longitudinal, prospective study on emotional adjustment before, during and after consecutive fertility treatment cycles	10.1093/HUMREP/DEI015	17
GAMEIRO S, (29), HUM REPROD UPDATE, V18, P652	Why do patients discontinue fertility treatment? A systematic review of reasons and predictors of discontinuation in fertility treatment	10.1093/HUMUPD/DMS031	14
VERHAAK CM, (30), HUM REPROD UPDATE, V13, P27	Women’s emotional adjustment to IVF: a systematic review of 25 years of research	10.1093/HUMUPD/DML040	14
BOIVIN J, (31), HUM REPROD, V22, P1506	International estimates of infertility prevalence and treatment-seeking: potential need and demand for infertility medical care	10.1093/HUMREP/DEM046	13
SLADE P, (32), HUM REPROD, V12, P183	A prospective, longitudinal study of emotions and relationships in *in-vitro* fertilization treatment.	10.1093/HUMREP/12.1.183	13
EUGSTER A, (33), SOC SCI MED, V48, P575	Psychological aspects of *in vitro* fertilization: a review	10.1016/S0277-9536(98)00386-4	11
NEWTON CR, 34, FERTIL STERIL, V72, P54	The Fertility Problem Inventory: measuring perceived infertility-related stress	10.1016/S0015-0282(99)00164-8	11
BOIVIN J, (10), BMJ-BRIT MED J, V342	Emotional distress in infertile women and failure of assisted reproductive technologies: meta-analysis of prospective psychosocial studies	10.1136/BMJ.D223	10
COUSINEAU TM, (35), BEST PRACT RES CL OB, V21, P293	Psychological impact of infertility	10.1016/J.BPOBGYN.2006.12.003	10
GREIL AL, (36), SOCIOL HEALTH ILL, V32, P140	The experience of infertility: a review of recent literature	10.1111/J.1467-9566.2009.01213.X	10
JORDAN C, (37), J BEHAV MED, V22, P341	Gender differences in coping with infertility: a meta-analysis	10.1023/A:1018774019232	10
VERHAAK CM, (8), HUM REPROD, V25, P1234	Who is at risk of emotional problems and how do you know? Screening of women going for IVF treatment	10.1093/HUMREP/DEQ054	10
BOIVIN J, (38), SOC SCI MED, V57, P2325	A review of psychosocial interventions in infertility	10.1016/S0277-9536(03)00138-2	9
BOIVIN J, (9), FERTIL STERIL, V83, P1745	Infertility-related stress in men and women predicts treatment outcome 1 year later	10.1016/J.FERTNSTERT.2004.12.039	9
OLIVIUS C, (39), FERTIL STERIL, V81, P258	Why do couples discontinue *in vitro* fertilization treatment? A cohort study	10.1016/J.FERTNSTERT.2003.06.029	9
ABBEY A, (40), PSYCHOL WOMEN QUART, V15, P295	Gender’s role in responses to infertility	10.1111/J.1471-6402.1991.TB00798.X	8
BOIVIN J, (41), FERTIL STERIL, V64, P802	Stress level across stages of *in vitro* fertilization in subsequently pregnant and nonpregnant women	10.1016/S0015-0282(16)57858-3	8
BOIVIN J, (42), HUM REPROD, V13, P3262	Psychological reactions during *in-vitro* fertilization: similar response pattern in husbands and wives.	10.1093/HUMREP/13.11.3262	8
BRANDES M (43), HUM REPROD, V24, P3127	When and why do subfertile couples discontinue their fertility care? A longitudinal cohort study in a secondary care subfertility population	10.1093/HUMREP/DEP340	8

### 3.7 Analysis of Keywords

**Figure 8:**
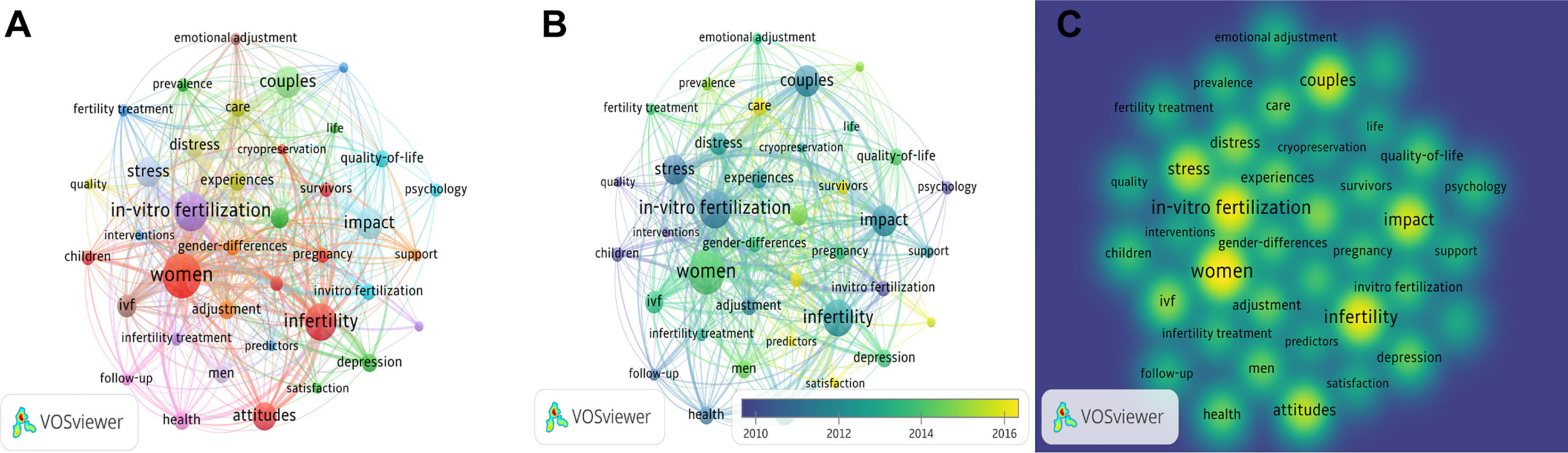
Co-occurrence analysis of keywords. **(A)** Mapping of keywords of studies. **(B)** VOSviewer overlay visualization of co-occurring author keywords by time(blue:earlier, yellow: later). **(C)** Distribution of keywords according to the mean frequency of appearance. The deeper the color of a node, the more frequently keywords appear.

**Figure 9:**
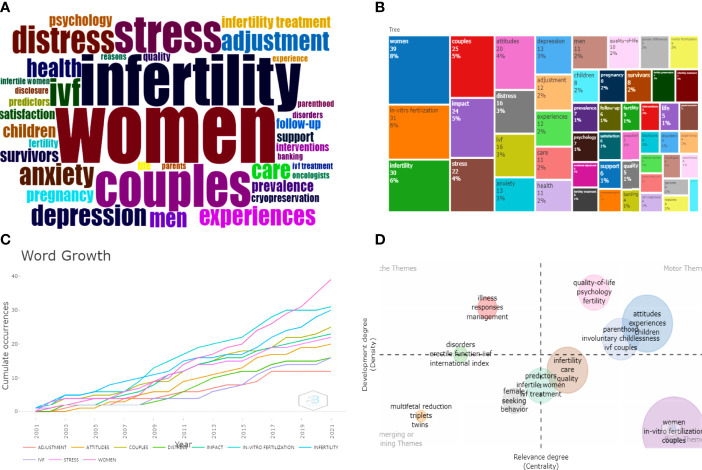
**(A, B)** Keywords cloud map and tree map related to infertility and psychology research. **(C)** Growth trends of the top 10 keywords in infertility and psychology research from 2001 to 2021. **(D)** Thematic Map of keywords of studies.First quadrant (upper right corner): motor themes; second quadrant (upper left corner): highly developed and isolated themes; third quadrant (lower left corner): emerging or declining themes; fourth quadrant (lower right corner): basic and transversal themes.

We analyzed a total of 38 keywords among 452 keywords related to infertility and applied research on psychological topics that were identified as having occurred more than five times (**Figure 8A**). The colors in the overlay visualization shown in **Figure 8B** indicate the average publication year of the identified keywords. Most of the keywords were published after 2014, with greener or yellower colors. The density visualization showed the same identified keywords mapped by frequency of appearance (**Figure 8C**). The top 10 keywords were: women (39 times), *in-vitro* salt (31 times), infertility (30 times), couples (25 times), impact (24 times), stress (22 times), attitudes (20 times), distress (16 times), IVF (16 times), and anxiety (1). Through the co-occurrence analysis of 452 keywords, the development trend and strategic coordinate map (**Figure 9**) were drawn. It can be seen that the covered groups with psychological problems of infertility mainly existed in women and in both husbands and wives, and their negative emotions were mainly stress, distress, anxiety, and depression. IVF was most likely to cause these negative emotions during treatment, and finding a solution often depended on the patient’s attitude, which would also have an impact on the outcome of subsequent treatment (**Figures 9A, B**). The frequency of the above top 10 keywords increased with time (**Figure 9C**). The keyword “women” grew the fastest, followed by “*in-vitro* fertilization.” Through the strategic coordinate chart of keywords (**Figure 9D**), quality of life, psychological status, fertility, attitude, experience, and IVF couples were major themes; illness, responses, and management were highly developed and isolated themes; multifetal reduction, triplets, twins, female, and seeking behavior were emerging or declining themes; and women, *in-vitro* fertilization, and couples were basic and transversal themes.

## 4 Discussion

In the present study, we combined bibliometric analyses with network visualizations to characterize the current landscape of infertility and psychology; analyzing the contributions of countries, institutions, journals, and authors to this emerging field; and predicting hot topics that will be of continued research interest in the coming years. Bibliometric analysis is a popular and rigorous method for exploring and analyzing large volumes of scientific data. Indeed, the enhanced understanding of science through bibliometric analysis can Not only discover the current state of research in this field (through numerical and image visualization) but also facilitate knowledge creation and shed light on the emerging areas in that field. From the above data, we found that infertility has a close relationship with psychology, which has attracted more and more attention in the follow-up diagnosis and treatment. Through the analysis of the above data, we summarized and thought about it.

### 4.1 General Trends in the Research of Infertility and Psychology

Since the field emerged in 2001, its annual publication output has increased in a volatile fashion. The publication output in 2012 was the highest, accounting for 8.11% of all the included studies. It can be seen that psychology has attracted more and more attention in infertility, and various countries have begun to analyze the psychology of patients with infertility. The reason why psychology attracts more and more attention to infertility is that, on the one hand, the development of assisted reproductive technology is more and more mature and the patients’ psychology is concerned. For example, there are several articles published in 2012 related to *in vitro* fertilization, including *Feasibility of Screening Patients for Emotional Risk Factors before In Vitro Fertilization in Daily Clinical Practice: A Process Evaluation* and *Perspectives on Access to In Vitro Fertilization in Portugal*. On the other hand, the reason may be a change in the model of modern medicine. Psychogenic diseases caused by various psychological and social causes have occurred widely. The modern medical model has been transformed into the “Bio-Psycho-Social Medical Model”, and psychological factors have been paid more and more attention.

### 4.2 Countries and Institutions That Influence the Direction of Research in This Field

With the largest number of publications and citations and the top rank for co-authorship analysis by country, the United Kingdom is currently the world leader in the research of infertility and psychology. According to the analysis of publishing institutions, the first and second universities in publishing volume were from the United Kingdom, namely Cardiff University and the University of Sheffield. These two universities have very strong comprehensive strengths. Cardiff University was the most productive and was ranked first in co-authorship analyses conducted by the institution, suggesting its close cooperation with other institutions. The Faculty of Biomedical and Life Sciences is Cardiff University’s largest faculty, and the Department of Psychology is ranked 2nd in the UK university Research Strength Rankings (REF). As one of the red brick universities, six Nobel Prize winners have graduated from the University of Sheffield or have long worked for the university’s researchers. These results suggest that the United Kingdom, especially the two universities mentioned above, may significantly impact the direction of research in this field and participate in the strongest collaborations worldwide. In the follow-up research, we can strengthen the cooperation with the UK and other countries, which can further explore the role of psychology in infertility.

### 4.3 A Pioneer in the Field of Infertility and Psychology

Boivin J, a doctor of psychology from Cardiff University, is a pioneer in the field of infertility and psychology. As a member of Cardiff Fertility Studies Research Group, he has studied the psychological aspects of infertility and had the largest number of publications (16, 10.60%) and citations (cited 890 times), and was the top rank for h-index and co-authorship analysis conducted. In a large Internet survey of 426 women from different countries about infertility counseling, he found that 56% had not consulted a doctor. Seeking medical advice for fertility problems is mainly associated with what women know or want to know about their fertility and their emotional health reactions to that knowledge. Negative reactions can substantially delay seeking help in 20% of women (17). He provided the guidelines for counseling in infertility about the purpose, objective, and typical issues and communication skills involved in psychosocial care to individuals using fertility services (15), and gave specific recommendations for infertility counselors in mental health and medical settings (44). He also proposed the idea that FertiQol is a reliable and sensitive measurement tool for quality of life in individuals with fertility problems, which provides an evaluation tool for future research on the psychological problems of infertile patients (10). He worked most closely with Schmidt L and Gameiro S, who are from the Institute of Public Health and have focused on the problems of both medical- and patient-centered (psychosocial) services (20, 25, 45). They have also suggested that men suffer from depression and stress during infertility (9, 11).

### 4.4 Hot Topics of Psychological Research on Infertile Patients in the Future

Keywords are the core of literature. They present the content of the whole article to the readers in the clearest way. By analyzing the composition, distribution, and clustering of keywords, the hot spot distribution in this field can be more quickly and effectively understood. The words “women,” “*in-vitro* fertilization,” “infertility,” and “couples” were the most frequently used keywords. This suggests that such psychological problems are widespread in women and more likely to occur during IVF treatment. Whether physical (e.g., failed treatment cycles, miscarriage, or fetal death) or emotional (e.g., loss of identity as a parent by never becoming pregnant), infertility diagnosis and the assisted human reproductive treatment process can have an impact on the emotional and mental health of couples. Through the strategic coordinate chart of keywords, we can see that the focus on infertility and psychology changed, for example, the quality of life, psychological status, fertility, attitude, experience, and the use of the keyword “IVF couples.” These words will be hot topics of psychological research on infertile patients in the future.

**Causes and objects of negative infertility psychology:** In terms of references cited, most of the top 20 references were reviews on the psychological aspects of infertility (27, 30, 46, 47), causes of negative psychology (48), and methods of emotional regulation (28, 31). Kitzinger C et al., who ranked first in global citations, clarified the causes of infertile patients’ negative psychology and psychological intervention from the perspective of PCOS (26). From PCOS and IVF, with the progress of medical technology and the development of new technologies, more factors affect infertile patients’ psychology. Greil AL from 1997 and SOC SCI MED, the most widely referenced source, focused specifically on the relationship between gender and infertility experience early on, noting that men also suffer from negative emotions (such as depression and anxiety) during infertility treatment. The research range of psychological factors of infertility has changed from female to male and female. Stress factors are the primary cause of negative psychology in infertile patients (27).

**Treatment to improve the negative psychology of infertility patients:** Researchers have found that improving the quality of life and fertility preservation is of utmost importance to ameliorate negative psychology in infertile patients (49). The patient’s attitude and experience play a crucial role in infertility psychological diagnosis and treatment, and positive psychotherapy is innovative for treating psychological disorders and enhancing positive emotions (50). When comparing the psychosocial characteristics of IVF-ET couples and couples without infertility, some scholars found that regardless of gender, the former had a significantly higher risk of anxiety and depression than the latter (51). Research has shown that 23% of patients end treatment prematurely because of perceived treatment burden (43). One-third of patients end treatment before pregnancy has been achieved (52). Anxiety about the viability and health of the fetus increased even after a successful pregnancy (21). Their anxiety and depression also increased with the embryo transfer (FET) process (53), which is in line with the ESHRE guidelines (54), stating that patient emotional stress peaks during the waiting period before FET and pregnancy testing. SCREENIVF can be used as a screening tool to identify women with a risk profile for emotional problems and is most effectively used in fertility clinics at the start of treatment in clinical practice (8, 55). In the psychological intervention of infertile patients with negative psychology, it is necessary to pay more attention to the psychological problem, put the patient at the center of nursing care, and view patients, the industry, and fertility clinics as all involved in delivering psychosocial care before, during, and after treatment (56).

It can be seen from the keyword analysis chart that the focus of attention has gradually shifted from the audience to the treatment methods and care (57–59). Currently, there is a lack of psychological coping plans and guidelines for infertility. Most of the literature is related to the treatment and care of infertility. Psychological support before, during, and after infertility treatment is critical for managing the psychosocial aspects of infertile couples’ grief responses. From a psychological perspective, grief in infertile women experiencing infertility treatment, whether it is caused by several physical examinations or pregnancy failure, will make it difficult to adhere to subsequent infertility treatment. Psychological interventions can be defined as a set of abilities that enable individuals to better adapt to challenging situations psychologically (60, 61). There are two main types of treatment: one based on mind-body intervention or relaxation therapy, and the other based on cognitive behavioral therapy, including understanding the cognitive situation, cognitive remodeling, and relaxation training. At the same time, various scales can be used to evaluate the patient’s psychological state, and psychological counseling or relaxation treatment can be carried out based on the cause.

## 5 The Research Status and Future Outlook

From our findings, there is a strong link between psychology and infertility. Psychology has been paid more and more attention to infertility, but it has not formed a systematic theory on the research contained within the past two decades (2001–2021). As for the psychological characteristics, causes, diagnosis and treatment methods of infertility, we will make a systematic review of the current research status and provide suggestions and reflections for future research in the next sections.

### 5.1 Factors Affecting the Psychological Status of Infertile Patients

#### 5.1.1 Female Dimension

As the subject of conception, women’s psychological state directly impacts the treatment process. This psychological state is often related to various factors, which can be summarized as “intraindividual,” “situational,” and “interpersonal.”

Intraindividual factors include age, years of marriage (years of infertility), BMI, living area, personality factors, and attitudes. Age and time of infertility are related to the ART pregnancy rate (51). An older age and a longer period of infertility, coupled with a personality that is easily influenced by others, will suffer greater psychological pressure during infertility diagnosis and treatment. Purewal found in a systematic review and meta-analysis that lifestyle (smoking, alcohol consumption) and body mass index (BMI) are predictors of a successful outcome for ART treatment and that obesity negatively affects ART outcomes (62). Anxiety and depression are often associated with obesity and binge eating (63, 64). In addition, the individual’s educational level, understanding, and processing of disease or treatment information are also related to the smoothness of the infertility treatment process. The lack of awareness of new technologies and the influence of others may impact their tolerance to fertility treatment. Expectations can cause psychological pressure (65, 66). Women with DOR had statistically significantly higher infertility distress scores (67).

Situational factors include economic status, treatment costs, and social pressure, which affect the psychological state of infertile patients. In assisted reproduction, *in vitro* fertilization, egg freezing, and pre-embryo transfer genetic testing (PGT) require expensive fertility treatment costs, especially after repeated attempts after conception fails, which can increase the economic burden on the patient’s family, causing the patient to suffer mental stress (65, 68). In addition, social factors also play a role, such as anxiety, tension, and worry about the interruption of infertility treatment during the COVID-19 pandemic. The study also found that during infertility treatment, multiple blood tests or injections of drugs, *in vitro* fertilization, or egg freezing, the waiting period and expected results undoubtedly aggravated the patient’s psychological pressure, affected their mental health, and further affected the treatment results (69).

Interpersonal factors include excessive concern from relatives and friends and increased disharmony in marital relations, which are signs of anxiety and depression in infertile couples. Reducing emotional distress and uncertainty can improve patients’ well-being during childbearing. Difficulties between partners in communicating are also predictors of high fertility stress (45). This tension and stress in the relationship affect the success of treatment (9). Schmidt, L et al. surveyed a large sample of 1934 patients with a 12-month data survey on satisfaction after receiving medical care and patient-centered services and found that the marital relationship was a significant factor affecting fertility treatment and obtaining the spouse’s support to meet emotional needs was conducive to adherence to follow-up treatment (20).

#### 5.1.2 Male Dimension

The mental health of male infertile patients should not be ignored, as mental health also has a serious impact on men. In Jessica S. Flynn’s study (70), more than half of the 138 parents preferred their sons to freeze their sperm, as evidenced by parental social pressure (71). Many studies have suggested that depression is as common in infertile men as in infertile women (72–75). The study by Gorkem Tuncay et al. determined that in couples, both parties had moderate-to-high levels of psychological stress during ART (76). Men’s age, income, causes of infertility, prognosis, duration of infertility, treatment costs, and perceived social support can all affect the male partner’s mood, and the physical and psychological burdens generated during diagnosis and treatment can affect the couple’s life quality (9). Katarzyna’s study found that ongoing infertility treatment could reduce the social functioning of male partners, leading to decreased perceived social support, increased social isolation, and an inability to meet social expectations associated with pregnancy (77). Such shame, guilt, and confusion toward parents, wives, colleagues, and even marriage will lead to secrecy about the disease and communication barriers to follow-up treatment. Such a vicious circle makes it difficult to take effective treatment measures for infertility cases.

However, some scholars have found that psychological conditions have little effect on the results of infertility treatment, possibly due to the small sample size (78, 79). When measuring psychological conditions, psychological scales such as anxiety and depression scales or instruments for measuring stress are often used, subject to a certain degree of subjectivity, but, understandably, these scales can evaluate negative emotions. Therefore, it is necessary to improve the quality of psychological research to explore the correlation between infertility and psychology better. Research in this area, such as the management of stress and the intervention of evaluating the results, is in progress.

### 5.2 Psychological Factors in the Etiology of Infertility

Studies on the role of psychological factors in the etiology of infertility have not yet provided unique data and require further investigation (80). From the perspective of psychological stress theory, external factors include events such as self-anxiety, parental pressure, and complaints, which lead to long-term negative emotions, such as anxiety, depression, and low self-esteem in patients, which constitute psychological stress for patients, and have deleterious effects on the reproductive system, causing subsequent pregnancy failure (81–83). The source of stress can cause the patient to produce a stress response, thereby generating neurotransmitters (such as catecholamines, including dopamine and norepinephrine) that affect the hypothalamic-pituitary axis (84, 85). They can affect the secretion of LH by affecting the release of gonadotropin-releasing hormone (GnRH), resulting in an imbalance of reproductive endocrine secretions, which in turn affects fertility. Studies have shown that chronic psychological stress can lead to endocrine diseases such as PCOS, premature ovarian failure, and infertility in women; it can also directly change the frequency pattern of the GnRH release in the hypothalamus resulting in relative infertility (86, 87). A specification published by the American Society for Reproductive Medicine and the American Society for Reproductive Endocrinology in 2017 states that psychological stress is a major obstacle to sexual function and fertility (88).

In addition, under psychological stress, the activated hypothalamic-pituitary axis will also promote the secretion of the corticotropin-releasing hormone (CRH), thereby increasing the release of the adrenocortical hormone (ACTH) and inducing the increase of glucocorticoids (such as cortisol) (76, 89), possibly directly and indirectly leading to a reduction of GnRH levels and the secretion of downstream hormones such as FSH and LH, which in turn affect ovarian function. In particular, the enhanced secretion of cortisol is a key factor in the stress response and one of the mediators of stress-induced inhibition of the reproductive system. Glucocorticoids have effects on the ovary, uterus, and testes. They directly affect granulosa cells and oocytes, and the mechanism may be that corticosterone causes concentration-dependent activation of oocyte extracellular signal-regulated kinase 1/2 and prevents blastocyst development (90). Furthermore, glucocorticoids (such as cortisol) stimulate mitochondria and then affect oocyte development or cause granulosa cell death, resulting in decreased reproductive function (87, 91–93). Changes in glucocorticoids under psychological stress in the uterus may affect fertility through gene expression changes, endometrial growth inhibition, or estrogen antagonism. For male reproductive function, psychological stress such as anxiety, depression, and negativity, including psychological stress during sperm retrieval, can produce high levels of glucocorticoids, directly inhibit the function of the testes, and inhibit the production and maturation of sperm (57, 94).

### 5.3 Psychological Management of Infertility

#### 5.3.1 Doctor-Patient Communication

Health care professionals play an important role in the diagnosis and treatment of infertility. Infertility and treatment are hard to control, and chronic stressors that can have severe, long-lasting negative social and psychological consequences. A diagnosis of infertility, regarded as a “symbolic loss,” is one of the causes of grief for those affected. This symbolic loss cannot be clearly identified, and it is hard to publicly express and relieve the sense of loss (57). Infertile patients’ lack of trust in medical staff not only affects their own emotions but also affects doctors to a certain extent, causing difficulties in treatment. Doctors’ progressive defensive retreat and detached attitude toward the disease will make patients feel more depressed and anxious and make the doctor treat them negatively. The negative interaction between the two sides will form a vicious circle, affecting the fertility results.

The European Society for Human Reproduction and Embryology (ESHRE) guidelines (54) point out that doctors should constantly exchange relevant diagnosis and treatment information during diagnosis and treatment to reduce psychological pressure and anxiety in the process and pay attention to and deal with patients’ psychological conditions before, during, and after treatment. They should consider the patients’ status and psychosocial needs, including behavioral (lifestyle, exercise, nutrition, and compliance), relational (relationship with partner (if there is one), family, friends, and a larger network, as well as their work), emotional (well-being, e.g., anxiety, depression, and quality of life), and cognitive factors (treatment concerns and knowledge). Poor psychological management is an important reason for interrupting infertility treatment (46). At the same time, patients’ lack of education and lack of treatment cognition will also reduce patients’ expectations of fertility treatment. Therefore, it is emphasized to strengthen psychological management in infertility treatment and have a protocol with thorough psychological assessment in a systematic and structured way (95). Particularly in treatment, including new technologies such as egg freezing *in vitro* fertilization and pursuing preimplantation genetic testing (PGT), it is necessary to provide professional technical guidance and psychological services for patients as well as help patients deal with their emotions, find coping strategies, and solve stressors (25). After infertility diagnosis and subsequent ART failure, more attention should be paid to psychological conditions, the importance of improving empathy and active listening, and specific psychological interventions that help reduce stress and improve couples’ well-being. An attempt should be made to strengthen the three dimensions of patient-centered care (PCC), which are communication, information, and continuity of care (96). Improving communication quality is the key to improving patient coping ability, well-being, treatment compliance, and continuous care.

Medical staff must adjust their own psychological states, recognize the intractability of infertility, strengthen communication between doctors and patients, and strengthen inter-professional and multi-disciplinary treatment by gynecologists, nurses, midwives, embryologists, and psychologists as part of a multi-disciplinary team of physicians. Other professionals can improve the quality of reproductive health care provided to patients with infertility, which can also help patients adhere to infertility treatment (96). It should be recognized that to achieve integrated care, mental health and medical professionals, as well as the industry, will need to work collaboratively and effectively to create new forms of psychosocial support for patients (56). A recent example of this type of successful collaboration is a Spanish project that improved the empathic skills of 13 fertility clinic staff through training from a psychologist (97). This project proved that it is possible and feasible for psychologists and physicians to work together to improve patient care.

#### 5.3.2 Social Support

The psychological pressure on patients and partners comes from not only themselves but also family members. The broken words of partners and the attitude of medical staff also affect their mental health and the probability of pregnancy. Social support is particularly important, which is not only reflected in the support of tools (such as tangible items, money, or experimental treatment) or the support of information (such as popular science knowledge or multimedia education platforms) but also in emotional support, such as love and encouragement.

Numerous studies have demonstrated that social support has a protective effect on physical and mental health, especially emotional support, which may help protect IVF individuals’ mental health, help continue the treatment cycle, and increase the odds of a good outcome (98–100). In addition to cognitive behavioral therapy, which is described below, group psychotherapy mainly comes from individuals’ networks, such as family members, friends, medical institutions, medical staff, counselors, or other personnel. Studies have shown that multi-dimensional and multi-angle social support can improve mental health, including anxiety, depression, and emotional distress; improve the constant treatment rate of *in vitro* fertilization, and reduce the risk of pregnancy failure (101). In addition, social support also depends on the cooperation of the government, regulatory authorities, and various professional institutions to create a harmonious and healthy social environment for infertile patients to ensure the smooth development and continuation of treatment. In addition, it is crucial to choose the right counselor by understanding the individual/couple and their situation. Infertility counselors use the best methods to educate couples about medical, legal, and psychological issues regarding the concept of a third party. It can be beneficial to improve the quality of life of couples while also giving them more confidence in choosing treatment options (44, 58).

#### 5.3.3 Cognitive Behavioral Therapy

Cognitive-behavioral therapy (CBT) is a type of psychotherapy based on cognitive models. This therapy has been extensively tested and proven effective in various mental illnesses, psychological problems, and medical problems (58). For patients that need to undergo cognitive remodeling, it is necessary to understand the current treatment of infertility actively and the development of technology (such as the application of microfluidic technology in IVF\IUI\ICSI), and then carry out relaxation training and guide patients to choose daily relaxation methods according to their preferences, such as reading, painting, and aerobic exercise. Frederiksen Y (102) conducted a meta-analysis of 39 studies of psychosocial or psychophysiological interventions in infertile patients participating in ART for 36 years (1978–2014) and concluded that CBT and psychosomatic interventions may be beneficial in reducing psychological distress and improving assisted reproductive outcomes, and are especially suitable for women with primary infertility, multiple pregnancy failures (103), and marital dissatisfaction (104).

We should identify cognitive, affective, and behavioral strategies to effectively deal with infertility by emphasizing emotional expression and positive orientation. Some scholars have found that worse circumstances (such as breast cancer) will make patients more likely to accept fertility protection and be fully prepared for future childbearing, and the more relaxed they are, the more they can adhere to treatment and conceive successfully (103). Infertile patients can change their cognition, treat themselves well, try to control their emotions, and maintain a positive attitude and self-compassion, which can effectively alleviate the negative psychological effects of infertility. This is especially important for infertile patients (105).

#### 5.3.4 Physical Exercise and Relaxation Techniques

During psychological stress, changes in the activity of several neural and endocrine circuits may affect the physiology of the musculoskeletal system, resulting in increased muscle tone, which may be harmful to later fertility. Progressive muscle relaxation (PMR), as one of the methods of exercise, can inhibit the hypothalamic-pituitary-adrenal axis and reduce the release of excitatory hormones and neurotransmitters, thereby regulating the calcium ion concentration of skeletal muscle cells and improving muscle tone. The reticular structure (RF) mediates and reduces muscle tension, improves cerebral cortex and limbic system activity, and reduces stress (58). Aerobic exercise and yoga are examples of good choices for activities to relax the muscles. Deep breathing relieves emotional tension and regulates the nervous system (58). It has been particularly pointed out that infertility caused by polycystic ovary syndrome often presents endocrine disorders such as being overweight and hirsutism. Appropriate exercise to lose weight can also relieve stress, maintain the balance of endocrine hormones in the body, enhance patients’ self-confidence, and promote the return to normal ovulation function, thereby enhancing the chances of conception. In subsequent summaries of clinical trials and literature reviews (105, 106), it has been pointed out that the implementation of stress management and advocacy of complementary therapies (such as psychological and physiological interventions) is beneficial to patients’ psychosocial health and stress. In the treatment of infertility, the pressure of the male partner is not to be underestimated, so muscle relaxation interventions are also crucial for men.

### 5.4 Directions for Future Research

There are many reasons leading to infertility. Different psychological problems are easy to occur in each stage. Based on the current research status, we still need to further discuss the following contents in future studies.

(1)Identify psychological problems at each stage of infertility treatment: There are many negative psychosocial manifestations, including depression, despair or loss of hope, guilt, anger, anxiety, depression, pain, shock, and even suicidal thoughts and feelings of isolation. However, there was a slight difference in the negative psychological performance at different stages of infertility treatment. Follow-up studies need to be summarized in order to facilitate follow-up targeted treatment.

(2)Pay attention to the couple’s psychology and joint treatment: Both men and women can have psychological problems due to infertility. Previous studies were mostly based on the perspective of women. In the follow-up studies, we need more scholars to sum up, and think deeply about male psychology. Joint therapy can better relieve psychological anxiety. If necessary, pressure from friends and family on both sides should also be paid attention to.

(3)Conduct a multidisciplinary study on the factors affecting the psychological state of infertility: From the current research, most of the factors affecting the psychological state of infertility patients are discussed from the perspective of physiology and pathology. We can be further explored in combination with multiple disciplines so as to carry out follow-up targeted diagnosis and treatment.

(4)Develop a consensus on psychological assessment methods and treatment: Considering that there are many psychological assessment scales at present, a unified evaluation standard is needed to evaluate the psychological status of infertility patients in future studies, which is also one aspect that we need to think about and summarize. It also has guiding significance for subsequent diagnosis and treatment. More importantly, we need a unified consensus on the treatment of infertility patients’ psychological problems. In future studies, we need more clinical medical staff to participate in the evaluation, selection and innovation of current treatment methods so as to form a systematic diagnosis and treatment theory.

## 6 Conclusion

Relatively little attention has been paid to the psychological level of infertility. The psychological emotions of concern are mainly stress, distress, and anxiety. The pathogenesis for infertility is still unclear, and there is a lack of unified and recognized targeted treatment. The use of relaxation methods is not unified. The psychological management methods and the methods of evaluating psychological conditions are different, and researchers have not yet reached a consensus. Therefore, how to better pay attention to the psychological status of patients, systematically manage each intervention technology, and evaluate the efficacy qualitatively and quantitatively are future research directions. These include evaluating the existence of anxiety and depression and other psychosocial manifestations in assisted human reproduction. It may also help to interpret physiological changes in observations to obtain high-quality, evidence-based medical evidence for psychological care to support treatment during infertility.

In sum, the psychological status of infertility still needs to be further explored by scholars. Whether it is the etiology, pathogenesis, research methods, or psychological management methods, all need to be discussed and unified. At the same time, national and regional cooperation can be strengthened, and more research can be done on the psychological aspects of infertility.

## 7 Strengths and Limitations

This was the first bibliometric analysis to investigate the psychological factors underlying infertility to the best of our knowledge. We conducted a comprehensive literature survey to perform quantitative and qualitative analyses of the authors’ publication output and research quality in the bibliometrix package. We also used a well-known scientific metrics software tool (VOS viewer) to construct and visualize bibliometric networks through co-authorship, co-citation, and co-occurrence analyses. However, our analysis had some limitations. First, the total amount of literature was relatively small. A total of 151 kinds of literature were published over 20 years, and searches were mainly performed in the WoS database. It would be better to combine these results and other databases, such as PubMed and Scopus. However, it is worth noting that WoS is the most commonly used scientometrics database, and most bibliometrics software can recognize the format from WoS. Second, although we conducted literature searches without any language restrictions, we only researched the English literature of WoS. Third, the keyword analysis results may have been affected by incomplete keyword extraction, and we may have overlooked the latest research trends owing to the emergence of low-relevance keywords. Fourth, as the psychological status of infertility is an emerging and developing field of research, we may have underestimated the contributions of different analyses. Recently published studies were cited infrequently because of their low frequency, although some were published in high-quality journals.

## Data Availability Statement

The original contributions presented in the study are included in the article. Further inquiries can be directed to the corresponding authors.

## Author Contributions

LS and WX designed the study. HKZ, LS, RW, LC, JW, MT, HQ and MW collected the data. HKZ, LS and RW analyzed the data and drafted the manuscript. WX, LW and HFZ revised and approved the final version of the manuscript. All authors contributed to the article and approved the submitted version.

## Funding

This work was supported by grants from the National Natural Science Foundation of China(82004413), the Medical Scientific Research Project of Jiangsu Provincial Health Commission(Z2020020), the Projects For Science and Technology of Chinese Medicine of Jiangsu Province in 2019 (YB201959, YB2020063), the Science and Technology Development Plan of Suzhou (SYS2020055), the Zhangjiagang Health Talent Project(ZJGWSRC202001, ZJGWSRC202007, ZJGQNKJ202008), the Science and Technology Development Plan of Zhangjiagang(ZKS1928), the Special Funds of the Key Clinical Disease in Diagnosis and Treatment Technology of Suzhou in 2019 (LCZX201920).

## Conflict of Interest

The authors declare that the research was conducted in the absence of any commercial or financial relationships that could be construed as a potential conflict of interest.

## Publisher’s Note

All claims expressed in this article are solely those of the authors and do not necessarily represent those of their affiliated organizations, or those of the publisher, the editors and the reviewers. Any product that may be evaluated in this article, or claim that may be made by its manufacturer, is not guaranteed or endorsed by the publisher.
